# Development of Foxp3+ regulatory T cell: epigenetics and more…

**DOI:** 10.1186/1479-5876-9-S2-I4

**Published:** 2011-11-23

**Authors:** Jochen Hühn

**Affiliations:** 1 Experimental Immunology, Helmholtz Centre for Infection Research, Braunschweig, Germany

## 

Abstract not submitted for publication

